# Terminated Trials in the ClinicalTrials.gov Results Database: Evaluation of Availability of Primary Outcome Data and Reasons for Termination

**DOI:** 10.1371/journal.pone.0127242

**Published:** 2015-05-26

**Authors:** Rebecca J. Williams, Tony Tse, Katelyn DiPiazza, Deborah A. Zarin

**Affiliations:** 1 National Library of Medicine (NLM), National Institutes of Health (NIH), Department of Health and Human Services (DHHS), Bethesda, Maryland, United States of America; 2 Johns Hopkins Bloomberg School of Public Health, Baltimore, Maryland, United States of America; University Hospital Basel, SWITZERLAND

## Abstract

**Background:**

Clinical trials that end prematurely (or “terminate”) raise financial, ethical, and scientific concerns. The extent to which the results of such trials are disseminated and the reasons for termination have not been well characterized.

**Methods and Findings:**

A cross-sectional, descriptive study of terminated clinical trials posted on the ClinicalTrials.gov results database as of February 2013 was conducted. The main outcomes were to characterize the availability of primary outcome data on ClinicalTrials.gov and in the published literature and to identify the reasons for trial termination. Approximately 12% of trials with results posted on the ClinicalTrials.gov results database (905/7,646) were terminated. Most trials were terminated for reasons other than accumulated data from the trial (68%; 619/905), with an insufficient rate of accrual being the lead reason for termination among these trials (57%; 350/619). Of the remaining trials, 21% (193/905) were terminated based on data from the trial (findings of efficacy or toxicity) and 10% (93/905) did not specify a reason. Overall, data for a primary outcome measure were available on ClinicalTrials.gov and in the published literature for 72% (648/905) and 22% (198/905) of trials, respectively. Primary outcome data were reported on the ClinicalTrials.gov results database and in the published literature more frequently (91% and 46%, respectively) when the decision to terminate was based on data from the trial.

**Conclusions:**

Trials terminate for a variety of reasons, not all of which reflect failures in the process or an inability to achieve the intended goals. Primary outcome data were reported most often when termination was based on data from the trial. Further research is needed to identify best practices for disseminating the experience and data resulting from terminated trials in order to help ensure maximal societal benefit from the investments of trial participants and others involved with the study.

## Introduction

Clinical trials depend on the participation of volunteers and involve significant investments of human, physical, and financial resources. Given these investments, trials that end prematurely (or “terminate”) without meeting their intended goals raise financial, ethical, and scientific concerns. It has been noted that when trials terminate there are: (a) opportunity costs associated with resources that could have supported other endeavors; (b) ethical issues regarding the enrolled volunteers whose participation may not contribute to meaningful scientific knowledge; and (c) scientific issues related to the decision to terminate as well as the appropriate interpretation of results.[1–6] However, there are also important and valid ethical and scientific reasons that trials may be terminated. For example, terminating a trial because of toxicity or efficacy-related findings is an expected part of responsible research.[7] Much of the prior research quantifying the frequency of trial termination, however, has focused on trial conduct issues such as poor participant accrual in specific therapeutic areas[8–10] or particular research settings.[11, 12] More recently, clinical trial registries have been used to evaluate reasons for termination across a broader range of trials,[13] but less attention has been given to understanding the extent to which primary outcome data from terminated trials are currently disseminated in the ClinicalTrials.gov results database or the biomedical literature.[14] Providing access to a summary of the primary outcome data at the end of a terminated trial, particularly when termination is based on findings during the trial’s conduct, helps to fulfill the promise made to research participants that their participation will result in knowledge that will contribute to the medical evidence base. We aim to evaluate this important aspect of trial termination by summarizing the extent to which primary outcome data are reported among terminated trials with results posted on the ClinicalTrials.gov results database based on the reasons for termination.

The ClinicalTrials.gov results database is a structured online system that provides the public with access to summary results and registration information for completed or terminated clinical studies. It is a unique source of results information about terminated trials because of the requirement under the Food and Drug Administration Amendments Act of 2007 (FDAAA), for summary results of certain trials of approved drugs and devices to be submitted within one year of final collection of data for the primary outcome, whether the trial concluded according to the pre-specified protocol or was terminated.[15] Failure to submit results within the specified deadline may result in civil monetary penalties and/or withholding of Federal funding. While the law specifically defines which trials are required to report results, we estimate that about half of all results posted on the ClinicalTrials.gov results database are not subject to the law. Given both the legal requirement to submit results for terminated trials and the additional submissions received, the ClinicalTrials.gov results database is a rich resource for quantifying whether data for primary outcomes are being reported as part of the study results and for evaluating termination reasons.

The summary results information submitted by the sponsor consists of four scientific modules: Participant Flow, Baseline Characteristics, Outcome Measures, and Adverse Events. While each module is considered “required,” it is currently possible to submit results information for a terminated study without providing outcome measure data if there is a clear explanation for why data can’t be reported.[16] However, the extent to which sponsors are using this option for terminated trials posted on the ClinicalTrials.gov results database has not yet been quantified. Thus, we were interested in exploring whether primary outcome data were being reported when posting results and if reporting varied based on the reason for termination. In this study we evaluated terminated trials posted on the ClinicalTrials.gov results database to determine: (1) reasons for termination and (2) whether primary outcome data were reported on ClinicalTrials.gov or in the published literature. As a secondary aim we assessed the availability of primary outcome data based on the percentage of target enrollment achieved at trial termination.

## Methods

### Data Source

The National Library of Medicine at the National Institutes of Health has operated the ClinicalTrials.gov registry since its inception in February 2000, adding the results database in September 2008. Summary protocol information is initially submitted to the registry by the sponsor (or principal investigator) at trial initiation and posted as a study record on ClinicalTrials.gov. Summary results are subsequently submitted by the sponsor and added to the study record after trial completion. Data elements specify the information (required and optional) to be submitted to ClinicalTrials.gov; specific data elements used in this analysis are noted throughout with Title Case.[17, 18] Complete information on the rationale, process, and requirements for registration and results submission has been described elsewhere.[19–22]

### Study Sample

To categorize the reasons for termination and examine the relationship to the availability of primary outcome data, we identified clinical trials (i.e., interventional studies) posted on the ClinicalTrials.gov results database with an Overall Recruitment Status of terminated, and Enrollment of at least 1 participant. ClinicalTrials.gov defines “terminated” as occurring when “recruiting or enrolling participants has halted prematurely and will not resume; participants are no longer being examined or treated.”[17] Trials that end prior to enrolling any participants are considered “withdrawn.” We limited the sample to terminated trials posted on the ClinicalTrials.gov results database in order to quantify, among trials reporting results, the extent to which primary outcome data were disclosed.

### Categorization of Reasons for Termination

Two authors (KD and RJW) independently categorized information provided in the Why Study Stopped? data element, an optional free-text field (160 character limit) available since February 2007. Three major categories of reasons for trial termination were derived from schemes described in previous research[1, 8, 23]: (1) termination based on scientific data collected during the trial; (2) termination based on reasons other than scientific data collected during the trial (“other reasons”); and (3) termination reason not provided. If multiple reasons were provided, a determination was made about which one was considered primary. Termination based on scientific data was always considered to be a primary reason if more than one category of reason was provided. Trials in the “other reasons” category were further organized into sub-categories. Although the initial plan was to use sub-categories of efficacy or safety for trials that terminated based on data from the trial, both were commonly mentioned in the context of an overall assessment and it was therefore not always clear which factor was dominant (e.g., “the perceived risk-benefit ratio for individuals with early active RA”).[24] Differences in assigning major categories and sub-categories were resolved by consensus.

### Determination of the Availability of Primary Outcome Data

We first evaluated whether the results section of the ClinicalTrials.gov record for the terminated trial included data for a primary outcome. We then determined whether the terminated trial was published in a journal indexed by MEDLINE, based on methods established in previous research.[25] In the results section of the ClinicalTrials.gov record, we summed the Number of Participants Analyzed across Arms/Groups (i.e., analysis group) for each Primary Outcome Measure. If the total analyzed was greater than zero in any Primary Outcome Measure, then primary outcome data were considered to have been reported. Similarly, a trial was considered to have published results if a journal publication included the results of at least one primary outcome measure (“primary publication”). Publications were initially identified from the ClinicalTrials.gov record using citations provided by the sponsor or that were automatically linked using the MEDLINE-indexed NCT Number (ClinicalTrials.gov Identifier).[26] Two authors (KD and TT) also searched PubMed by matching key data elements from the ClinicalTrials.gov study record and the publication including Intervention Name, Condition or Focus of Study, Sponsor, and Study Design.[25] Publications identified were reviewed for the presence of data for a primary outcome. A final search for publications was completed in December 2013. The number of terminated trials with primary outcome data reported in the ClinicalTrials.gov results database and in MEDLINE-indexed publications was summarized with respect to the reason for trial termination.

### Characterization of Target Enrollment Achieved at Termination

To further characterize terminated trials and the extent to which primary outcome data were available, we calculated the percentage of target enrollment achieved. Enrollment is specified as “Anticipated” when initially registering a trial, updated throughout the lifecycle of a trial, and finally specified as “Actual” at trial completion. We defined target enrollment as the value first specified for Enrollment (available in the ClinicalTrials.gov Archive site). We thus excluded trials for which the target enrollment was not known because either (1) the trial was first registered after the Primary Completion Date (or date not provided) or the (2) Enrollment was first specified as Actual or “zero” participants. The total enrolled in the trial was defined, for the purposes of this analysis, as the total number of participants assigned to an Arm/Group (e.g., participants enrolled but not randomized were excluded from the total). There are two potential sources for this data in a ClinicalTrials.gov record, Actual Enrollment in the protocol section and the number of participants identified as Started in the Participant Flow module of the results section. When the values were not identical between these two data sources, two authors (KD and RJW) independently reviewed the record to determine the appropriate value for the analysis, resolving any differences by consensus. If a record did not contain a clear explanation for a discrepancy, the value calculated from the sum of the number started in the Participant Flow module was used.

All analyses conducted were descriptive, summarizing the data for terminated trials posted on the ClinicalTrials.gov results database. Because this study is only intended to reflect the experience of the ClinicalTrials.gov results database, we did not make any statistical inferences. All data were summarized and analyzed using Excel 2010 (Microsoft Corporation, Redmond, WA) and an internal tool based on the Essie search engine.[27]

## Results

**Fig 1:**
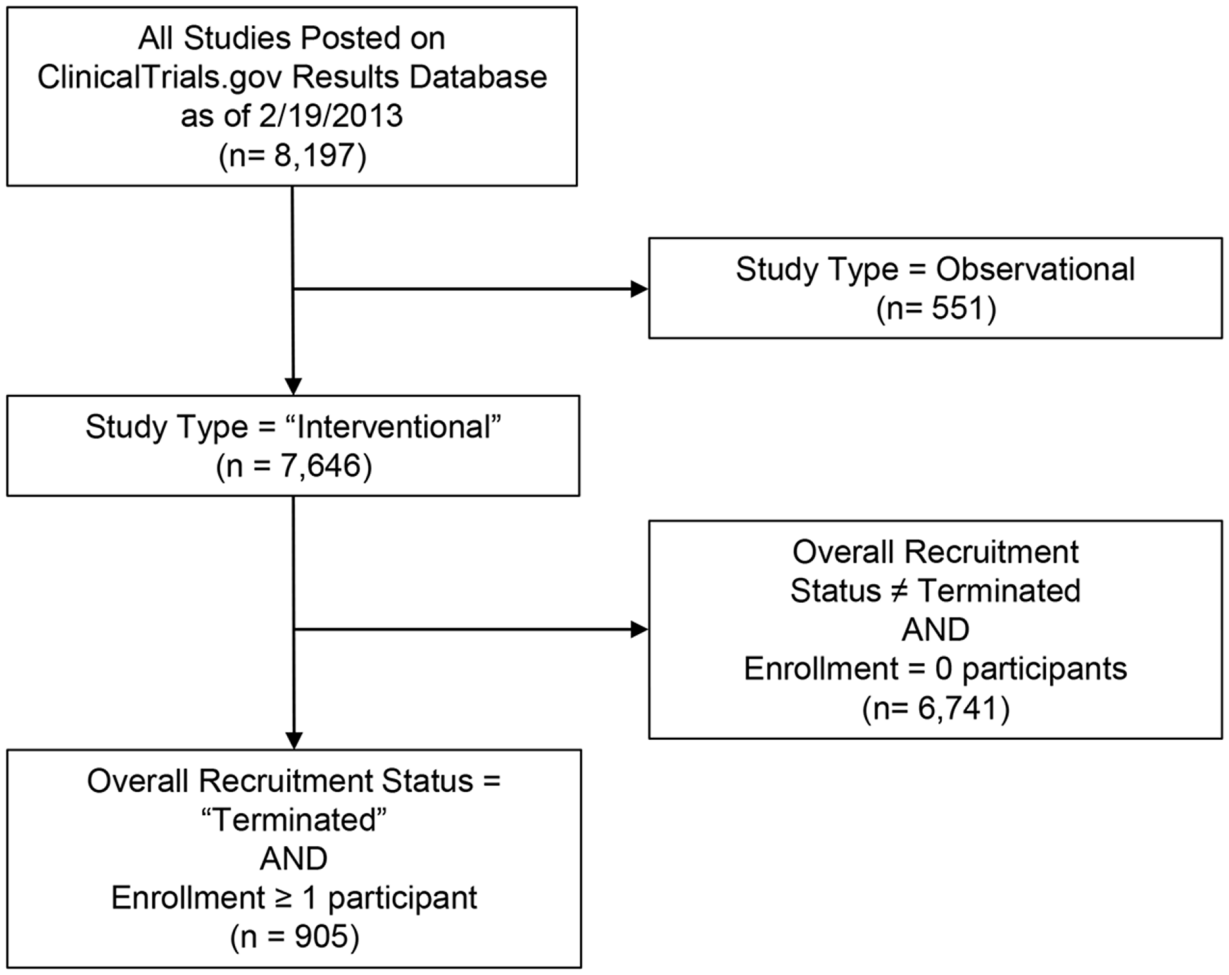
Study Inclusion Criteria. Presents ClinicalTrials.gov data elements and values used to filter records. Study Type of “Interventional” refers to clinical trials.

At the time of data collection, February 19, 2013, the ClinicalTrials.gov results database included 8,197 studies with results. There were 7,646 clinical trials posted on the ClinicalTrials.gov results database, of which 12% (n = 905) were listed as terminated and enrolled 1 or more participants (Fig 1). The characteristics of these trials are summarized in S1 and S2 Tables. Nearly all of the 905 trials had a Primary Completion Date of December 2011 or earlier (97%; 876/905), providing at least 2 years for reporting of primary outcome data.

### Reasons for Trial Termination

The majority (68%; 619/905) of trials with results were terminated for reasons other than scientific data from the trial (Table 1). Within this category, 11% (69/619) of the trials were terminated for reasons considered external to the trial (external information; product withdrawal) while the remaining 89% (550/619) of trials were terminated for reasons directly related to trial conduct or logistics. Insufficient rate of accrual was the leading sub-category, representing 57% (350/619) of trials terminated for other reasons and 39% (350/905) of terminated trials overall. Twenty-one percent (21%) of trials were terminated based on scientific data from the trial (e.g., findings related to the overall benefit-risk profile of the intervention(s) evaluated) and 10% of trials did not specify a reason for termination. The inter-rater agreement for categorizing the reasons for termination, calculated using Cohen’s kappa, was 0.86.

**Table 1:** Reason for termination categorization.

Termination Category	Number of Trials	Percentage (%) of Total Trials*
**Total Terminated**	**905**	**100%**
** 1. Scientific data from the trial**	**193**	**21.3%**
** 2. Other than scientific data from the trial**	**619**	**68.4%**
a. Insufficient accrual rate	350	56.5%
b. Unspecified business decision/strategic reason	77	12.4%
c. Trial administration or conduct (issues with protocol, investigators, site, etc.)	58	9.4%
d. External information (results from other trials, competing trials, or changes in standard of care)	51	8.2%
e. Funding	34	5.5%
f. Product withdrawal	18	2.9%
g. Lack of drug supply (other than drug withdrawal)	17	2.7%
h. Other (e.g., uninformative or non-specific text)	14	2.3%
** 3. Termination Reason Not Provided**	**93**	**10.3%**

*Sub-category percentages calculated as a percentage of all trials in Termination Category 2 (n = 619)

### Termination and Availability of Results Information

Overall, 72% (648/905) of terminated trials posted on the ClinicalTrials.gov results database reported data for a primary outcome, while 22% (198/905) had primary outcome data published in a biomedical journal (Table 2). Primary outcome data were reported on ClinicalTrials.gov more frequently when the decision to terminate was based on data from the trial as compared to other reasons (91% vs 65%). Similarly, trials were published more frequently when the trial terminated based on data from the trial as compared to other reasons (46% vs 14%).

**Table 2:** Termination categories and whether results for a primary outcome measure were (a) reported on ClinicalTrials.gov or (b) published in a biomedical journal (as of December 2013).

		ClinicalTrials.gov		Primary Publication	
Reason for Termination	Total # of Trials	YES N (% of Total)	NO N (% of Total)	YES N (% of Total)	NO N (% of Total)
**Scientific data from the trial**	193	175 (90.7%)	18 (9.3%)	88 (45.6%)	105 (54.4%)
**Other than scientific data from the trial**	619	402 (64.9%)	217 (35.1%)	86 (13.9%)	533 (86.1%)
**Reason not provided**	93	71 (76.3%)	22 (23.7%)	24 (25.8%)	69 (74.2%)
**Total**	**905**	**648 (71.6%)**	**257 (28.4%)**	**198 (21.9%)**	**707 (78.1%)**

### Target Enrollment and Availability of Results Information

**Fig 2:**
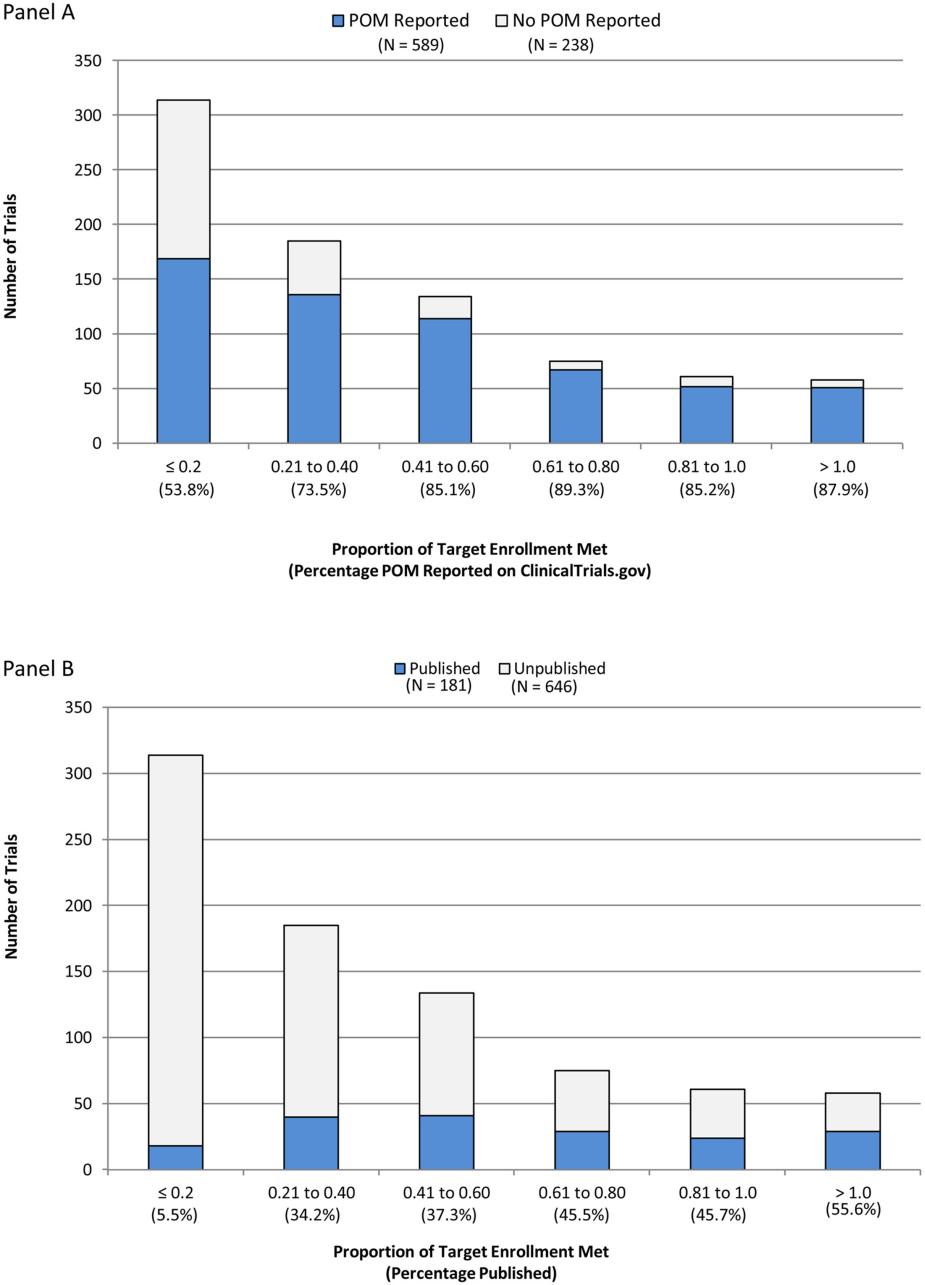
Number and Percentage of Terminated Trials by Proportion of Target Enrollment Met and whether results for a Primary Outcome Measure (POM) were (a) reported on ClinicalTrials.gov or (b) published in a biomedical journal (as of December 2013).

Of the 905 terminated trials on the ClinicalTrials.gov results database, 78 were excluded from analyses related to target enrollment achieved because the requisite information was not available (i.e., trial registered after completion or Anticipated Enrollment not specified). Of the remaining 827 trials, the median (interquartile range) target enrollment was 94 (IQR = 40–231) participants, while the median actual enrollment was 21 (IQR = 7–76) participants. The median percentage of target enrollment achieved was 30% (IQR = 12%- 58%) with 14% (119/827) of terminated trials achieving over 80% of target enrollment and 38% (314/827) achieving 20% or less of the target. As the percentage of target enrollment achieved increased, the frequency of reporting a primary outcome in ClinicalTrials.gov and in the published literature also increased (Fig 2). Trials achieving less than 20% of target enrollment had the lowest rates of reporting primary outcome data on ClinicalTrials.gov (54%; 169/314) and in the published literature (6%; 18/314). Overall, the ClinicalTrials.gov results database provided primary outcome data for 64% (415/646) of unpublished trials and 96% (174/181) of published trials.

## Discussion

We used the ClinicalTrials.gov results database to determine the availability of primary outcome data among terminated trials reporting results and the frequency and reasons for trial termination. Approximately 12% of all trials posted on the ClinicalTrials.gov results database were terminated, which is consistent with other cross-sectional studies using both the ClinicalTrials.gov registry [7, 9, 28] and funders’ administrative databases,[29] but lower than studies using ethics committee approvals as the primary data source.[14] Although our study sample is not generalizable to all clinical trials, we note that some differences in rates of termination in our sample versus that in other research studies may be due to the lack of a single established definition of terminated. For example, Kasenda et al. also relied on self-reports by investigators but from multiple sources and, if this information was not available, considered trials that achieved less than 90% of the target sample size to be “discontinued” (i.e., terminated). We relied solely on sponsor self-identification of a trial as terminated in ClinicalTrials.gov, and there may have been heterogeneity in how sponsors applied the ClinicalTrials.gov data element definition resulting in (1) the inclusion of trials that achieved their target enrollment but were considered by the sponsor to have ended prematurely (e.g., data collection ended early) and (2) the exclusion of trials that had lower than expected enrollment but were identified by the sponsor as “Completed”.

The information provided by sponsors describing the reason for termination was typically sufficient to allow for consistent categorization between reviewers. Most study records provided the reason in the Why Study Stopped? free-text field, although 7% (63/905) of records (mostly from a single sponsor) provided a lengthier description in the Detailed Description field and 10% of records did not provide a reason. Our process for categorization relied on the judgment of the authors and when multiple reasons for termination were mentioned (6%; 55/905), we identified one as primary using the method previously described (in which “scientific data from the trial” was given precedence over other reasons). This process could have led to some misclassifications. Providing a structured menu of options with a free-text field for explaining why a study terminated on ClinicalTrials.gov may allow for more uniform, accurate, and complete collection of reasons for termination as well as easier retrieval from the database.

Overall, approximately 60% (550/905) of trials were terminated because of trial conduct-related problems. There is currently significant interest in efforts to reduce the frequency of these problems, particularly with respect to participant recruitment.[30] However, our data also highlight the fact that not all terminated trials reflect a failure in the clinical trial process. Over 20% of trials were terminated based on data accumulated during the trial, suggesting that the risk-benefit ratio was thought to be unacceptable or so clear that additional data collection could not be justified. An additional 8% of trials were terminated because information external to the trial had changed the landscape in a way that made the trial inappropriate, infeasible, or irrelevant. Thus our data indicate up to 28% of trial terminations may reflect the system working as it should, after trial initiation, to ensure the appropriate management of trial participants and related scientific resources based on monitoring of study data. Although, it is possible that some terminations could have been avoided as reflected in observations made by Ioannidis with respect to discontinued surgical trials: "In some cases, discontinuation may be the best course of action. Trials that prove to be futile should clearly be discontinued. However, for most trials that are discontinued early, this could probably have been avoided with more careful study design and upfront consideration of the recruitment landscape before starting the trial.”[31]

We determined that primary outcome data were available for a substantial proportion of terminated trials posted on the ClinicalTrials.gov results database (72% in ClinicalTrials.gov and 22% in published literature). These rates were higher when trials terminated based on data from the trial (91% in ClinicalTrials.gov and 46% in published literature). There are no prior studies evaluating primary outcome data available on the ClinicalTrials.gov results database for terminated trials. One study observed a publication rate of 45% among terminated trials,[14] while the publication rate for all clinical trials is estimated to be 40–70%.[25, 32–36] We did not compare primary outcome data reported on ClinicalTrials.gov with those reported in primary publications, however recent studies have observed inconsistencies.[37–39] Whether similar trends are found for terminated trials is a question that may warrant additional research. The legal requirements for submitting results of terminated trials to ClinicalTrials.gov largely influenced the reporting of primary outcome data in our sample. The low rate of publication observed may reflect a decision by sponsors not to pursue publication once the findings were made available on ClinicalTrials.gov and/or the sponsor viewing the findings as being of little interest to a journal or the clinical community at large. This appeared to be particularly true for the over 300 trials that achieved 20% or less of the target enrollment. We did not evaluate the explanations provided for why primary outcome data were not reported to ClinicalTrials.gov, but this would also be an area for further research. Anecdotally, sponsors are reluctant to submit outcome data they believe to not be meaningful or to be inconsistent with the protocol (e.g., primary outcome data collection was only up to 6 months, when the protocol specified 2 years). In addition, some sponsors may not have aggregated and analyzed the collected data, particularly if enrollment was very low. This raises interesting issues about expectations for disseminating findings, even when outcome data deviate from the protocol (due to termination) or yield results that are difficult to interpret in the context of termination. We note that in an exploratory analysis, serious adverse event information was reported for all terminated trials with results (n = 905), with the exception of 10 trials that submitted results to ClinicalTrials.gov before the adverse event module became mandatory in September 2009.

In addition to reporting information on primary outcome(s) and observed adverse events, there may be qualitative information about trial conduct and recruitment efforts that could help future researchers learn from the prior experience of a terminated trial. Existing reporting guidelines such as the Consolidated Standards of Reporting Trials (CONSORT) Statement provide instructions for describing decisions related to trial termination, but do not address the extent to which data and other information from trials ending prematurely should be summarized.[40] The Notice of Proposed Rulemaking (NPRM) for FDAAA, issued for public comment on November 21, 2014, describes the requirements for results submission at ClinicalTrials.gov. The NPRM proposes that all collected data for a primary outcome must be reported (taking into account privacy concerns), even when the actual enrollment is much lower than the target enrollment.[41] In our sample, even when the proportion of target enrollment achieved was 20% or less, over half of the trials reported primary outcome data when reporting results at ClinicalTrials.gov.

Limitations of this research include our sample being biased towards trials that are required to have results submitted under FDAAA (i.e., generally, Phase 2–4 trials of FDA-approved drugs, biologics, or devices conducted in the US), although results may be submitted to ClinicalTrials.gov for any registered trial. We estimate that about half of the trials posted on the ClinicalTrials.gov results database are subject to FDAAA. Restricting our search to publications indexed in MEDLINE may have led to some published trials not being counted. All information in a record on ClinicalTrials.gov is provided by the sponsor and, although, automated and manual quality checks are conducted by ClinicalTrials.gov before posting information there may be undetected errors (e.g., a terminated trial being incorrectly specified as “Completed”) or differences in the manner in which sponsors interpret and complete the required and optional data elements.

## Conclusions

Trials terminate for a variety of reasons, not all of which reflect failures in the clinical trial process or an inability to achieve the intended goals. Trials with results posted on the ClinicalTrials.gov results database had primary outcome data reported on the database and the published literature more frequently when the reason for termination was based on data from the trial. Further research into the best approaches for analyzing and describing the experience and data resulting from terminated trials would help to ensure maximal societal benefit from the investments of trial participants and others in the study.

## Supporting Information

S1 Table Characteristics of terminated trials in the ClinicalTrials.gov results database. (DOCX)

S2 Table Disease/Condition categories of terminated trials in the ClinicalTrials.gov results database. (DOCX)
