# Supplementary material for: Terminated Trials in the ClinicalTrials.gov Results Database: Evaluation of Availability of Primary Outcome Data and Reasons for Termination
Source: PLoS One. 2015 May 26;10(5):e0127242. doi: 10.1371/journal.pone.0127242 (PMC4444136; doi:10.1371/journal.pone.0127242)
Supplement: S1 Table — (DOCX) [file pone.0127242.s001.docx]

**S1 Table. Characteristics of terminated trials in the ClinicalTrials.gov results database**

| **Characteristic** | **All Trials with Results**  **# Records (%)** | **Terminated Trials with Results**  **# Records (%)** |
| --- | --- | --- |
| **Total** | **7,646** | **905** |
| **Primary Purpose** | | |
| Treatment | 5,921 (77.4%) | 802 (88.6%) |
| Prevention | 775 (10.1%) | 46 (5.1%) |
| Missing | 408 (5.3%) | 13 (1.4%) |
| Other | 375 (4.9%) | 29 (3.2%) |
| Diagnostic | 167 (2.2%) | 15 (1.7%) |
| **Intervention Type** | | |
| Drug | 5,887 (77.0%) | 767 (84.8%) |
| Biological | 820 (10.7%) | 86 (9.5%) |
| Procedural | 322 (4.2%) | 80 (8.8%) |
| Device | 697 (9.1%) | 75 (8.3%) |
| Other | 469 (6.1%) | 61 (6.7%) |
| Radiation | 85 (1.1%) | 29 (3.2%) |
| Behavioral | 197 (2.6%) | 13 (1.4%) |
| Dietary supplement | 100 (1.3%) | 12 (1.3%) |
| Genetic | 25 (0.3%) | 5 (0.6%) |
| **Study Phase** | | |
| 0 or 1 | 625 (8.2%) | 30 (3.3%) |
| 2 (including 1/2) | 2,114 (27.6%) | 407 (45.0%) |
| 3 (including 2/3) | 2,597 (34.0%) | 213 (23.5%) |
| 4 | 1,361 (17.8%) | 145 (16.0%) |
| N/A | 949 (12.4%) | 110 (12.2%) |
| **Intervention Model** | | |
| Parallel | 4,671 (61.1%) | 503 (55.6%) |
| Single Group | 2,092 (27.4%) | 354 (39.1%) |
| Crossover | 793 (10.4%) | 36 (4.0%) |
| Factorial | 68 (0.9%) | 7 (0.8%) |
| Missing | 22 (0.3%) | 50 (5.5%) |
| **Allocation** | | |
| Randomized | 5,344 (69.9%) | 532 (58.8%) |
| Nonrandomized | 1,286 (16.8%) | 184 (20.3%) |
| Missing | 1,016 (13.3%) | 189 (20.9%) |
| **Blinding** | | |
| Open | 3,882 (50.8%) | 575 (63.5%) |
| Double blind | 3,187 (41.7%) | 293 (32.4%) |
| Single blind | 531 (6.9%) | 32 (3.5%) |
| Missing | 46 (0.6%) | 5 (0.6%) |

|  |  |  |
| --- | --- | --- |
|  |  |  |
| **Characteristic** | **All Trials with Results**  **# Records (%)** | **Terminated Trials with Results**  **# Records (%)** |
| **Total** | **7,646** | **905** |
| **Data Monitoring Committee (DMC)** | | |
| Not Have DMC | 4,081 (53.4%) | 427 (47.2%) |
| Has DMC | 1,916 (25.1%) | 346 (38.2%) |
| Missing | 1,649 (21.6%) | 132 (14.6%) |
| **Lead Sponsor Type** | | |
| Industry | 5,301 (69.3%) | 495 (54.7%) |
| Other (academic, non-profit) | 2,065 (27.0%) | 364 (40.2%) |
| NIH | 280 (3.7%) | 49 (5.4%) |
| **Single/Multiple Site** | | |
| Multiple site | 3,819 (49.9%) | 435 (48.1%) |
| Single site | 2,828 (37.0%) | 385 (42.5%) |
| Missing | 999 (13.1%) | 85 (9.4%) |
| **Location Country** | | |
| US Only | 3,487 (45.6%) | 532 (58.8%) |
| Non-US Only | 2,006 (26.2%) | 156 (17.2%) |
| US and Non-US | 1,154 (15.1%) | 132 (14.6%) |
| Missing | 999 (13.1%) | 85 (9.4%) |
| **# Participants (Enrollment)** | | |
| 0 -100 | 3,823 (50.0%) | 698 (77.1%) |
| 101 – 1000 | 3,327 (43.5%) | 188 (20.8%) |
| > 1000 | 496 (6.5%) | 19 (2.1%) |
| Missing | 0 (0.0%) | 0 |
