# Supplementary material for: Terminated Trials in the ClinicalTrials.gov Results Database: Evaluation of Availability of Primary Outcome Data and Reasons for Termination
Source: PLoS One. 2015 May 26;10(5):e0127242. doi: 10.1371/journal.pone.0127242 (PMC4444136; doi:10.1371/journal.pone.0127242)
Supplement: S2 Table — (DOCX) [file pone.0127242.s002.docx]

**S2 Table. Disease/Condition categories of terminated trials in the ClinicalTrials.gov results database**

| **Disease/Condition Category** | **All Trials with Results**  **# Records (%)** | **Terminated Trials with Results**  **# Records (%)** |
| --- | --- | --- |
| **Total*** | **7,646 (100%)** | **905 (100%)** |
| Bacterial and Fungal Diseases | 839 (11.0%) | 72 (8.0%) |
| Behaviors and Mental Disorders | 843 (11.0%) | 84 (9.3%) |
| Blood and Lymph Conditions | 485 (6.3%) | 127 (14.0%) |
| Cancers and Other Neoplasms | 1,556 (20.4%) | 336 (37.1%) |
| Digestive System Diseases | 631 (8.3%) | 99 (10.9%) |
| Diseases and Abnormalities at or before Birth | 384 (5.0%) | 72 (8.0%) |
| Ear, Nose, and Throat Diseases | 198 (2.6%) | 14 (1.5%) |
| Eye Diseases | 350 (4.6%) | 19 (2.1%) |
| Gland and Hormone Related Diseases | 673 (8.8%) | 79 (8.7%) |
| Heart and Blood Diseases | 918 (12.0%) | 125 (13.8%) |
| Immune System Diseases | 1,041 (13.6%) | 137 (15.1%) |
| Mouth and Tooth Diseases | 125 (1.6%) | 10 (1.1%) |
| Muscle, Bone, and Cartilage Diseases | 522 (6.8%) | 62 (6.9%) |
| Nervous System Diseases | 1,333 (17.4%) | 172 (19.0%) |
| Nutritional and Metabolic Diseases | 840 (11.0%) | 69 (7.6%) |
| Respiratory Tract (Lung and Bronchial) Diseases | 983 (12.9%) | 127 (14.0%) |
| Skin and Connective Tissue Diseases | 981 (12.8%) | 138 (15.2%) |
| Substance Related Disorders | 132 (1.7%) | 16 (1.8%) |
| Urinary Tract, Sexual Organs, and Pregnancy Conditions | 835 (10.9%) | 137 (15.1%) |
| Viral Diseases | 648 (8.5%) | 55 (6.1%) |
| Wounds and Injuries | 141 (1.8%) | 29 (3.2%) |
| Missing | 711 (9.3%) | 38 (4.2%) |

*A trial may involve more than one Disease/Condition Category, therefore, the # of records is not additive.
